# Synthesis and Biological Activity of Organothiophosphoryl Polyoxotungstates

**DOI:** 10.1155/MBD.2002.257

**Published:** 2002

**Authors:** Zhengang Sun, Jutao Liu, Jianfang Ma, Jingfu Liu

**Affiliations:** 1 Department of Chemistry Northeast Normal University Changchun 130024 China

## Abstract

Organothiophosphoryl polyoxotungstates R_∋_XW_∞∞_O_∋∃_^/-^ , R_∋_ P_∋_W_∞_,O_∞_^/-^, R_∋_PW_∃_O_∋_ _Δ_^-^(X = P, Si, Ge, B or Ga; R = PhP(S), C_6_H_11_P(S)) have been prepared from lacunary polyoxoanions and PhP(S). The products were characterized by elemental analysis, IR, and NMR spectroscopy. According to spectroscopic observations, the hybrid anions consist of a lacunary anion framework on which are grafted two equivalent or groups through P-O-W bridges. Some of the title compounds showed the antigerm activity.


### SYNTHESIS AND BIOLOGICAL ACTIVITY OF ORGANOTHIOPHOSPHORYL POLYOXOTUNGSTATES

Zhengang Sun, Jutao Liu, Jianfang Ma, and Jingfu Liu*

Department of Chemistry, Northeast Normal University, Changchun 130024, P. R. China
<sdjingfu@public.cc.jl.cn>

### ABSTRACT

Organothiophosphoryl polyoxotungstates $R_2XW_{11}O_{39}^{n-}$, $R_2P_2W_{17}O_{61}^{6-}$, $R_2PW_9O_{34}^{5-}$, (X=P, Si, Ge, B or Ga; R=PhP(S), $C_6H_{11}P(S)$) have been prepared from lacunary polyoxoanions and $PhP(S)Cl_2$ or $C_6H_{11}P(S)Cl_2$. The products were characterized by elemental analysis, IR, $^{31}P$ and $^{183}W$ NMR spectroscopy. According to spectroscopic observations, the hybrid anions consist of a lacunary anion framework on which are grafted two equivalent $C_6H_5P(S)$ or $C_6H_{11}P(S)$ groups through P-O-W bridges. Some of the title compounds showed the antigerm activity.

### 1.INTRODUCTION

Polyoxometalates (POMs) are early transition metal oxygen anion clusters. Only more recently have some of the biological and pharmacologic properties of POMs been investigated [1]. The principal advantageous feature of POMs is that nearly every molecular property that impacts the recognition and reactivity of POMs with target biological macromolecules can be altered. Pendent organic biological groups could be used to increase recognition of key substructures in target biomacromolecules, and enhance the facility of drug formulation. The reactivity of lacunary polyoxometalates with organic and organometallic groups has been summarized [2]. To date, the reaction of lacunary polyoxotungstates with organophosphonic acid has been reported rarely, except for a unique study of Kim and Hill [3] on PhPO derivatives of monovacant tungsto-phosphate and -silicate, and Thoucenot on RPO derivatives of trivacant tungsto-phosphate and divacant tungsto-silicate [4]. There are only two or three papers involving the biological properties of POMs derivatized with organic groups. In order to develop this uncharted territory, we investigated the biological activity of the organotin, organotitanium and organophosphory polyoxotungstates [5-8]. We report herein the preparation and biological activity of organophosphoryl polyoxotungstates $R_2XW_{11}O_{39}^{n-}$, $R_2P_2W_{17}O_{61}^{6-}$ and $R_2PW_9O_{34}^{5-}$ (X=P, Si, Ge, B, Ga; R=PhP(S), $C_6H_{11}P(S)$ ).

### 2. EXPERIMENTAL

### 2.1 Material

All reagents were of analytical or guaranteed grade; MeCN was distilled over $P_2O_5$, and used immediately. $PhP(S)Cl_2$ and $C_6H_{11}P(S)Cl_2$ were prepared following literature procedures[9,10]. $Na_7PW_{11}O_{39}\cdot 13H_2O$[11], $K_6Na_2SiW_{11}O_{39}\cdot 13H_2O$[12], $K_6Na_2GeW_{11}O_{39}\cdot 13H_2O$[12], $K_7NaBW_{11}O_{39}\cdot 13H_2O$[12], $K_9GaW_{11}O_{39}\cdot 13H_2O$[11], $K_{10}P_2W_{17}O_{61}\cdot 17H_2O$[12] and $Na_8HPW_9O_{34}\cdot 24H_2O$[13] were prepared using procedures described in the literature. Their purity was checked by I R or $^{31}P$ NMR spectroscopy.

### 2.2 Preparation of compounds

#### 2.2.1 $[Bu_4N]_2H[PhP(S)]_2PW_{11}O_{39}$ **1**

$[Bu_4N]_4H_3PW_{11}O_{39}$ (1.83 g, 0.5 mmol) was dissolved in MeCN (20 ml), to which was added dropwise $PhP(S)Cl_2$ (1 mmol) in MeCN (10 ml) with rigorous stirring, and the mixture was stirred for 48 h at room temperature. After separation of a white solid, the resulting brown yellow solution was concentrated in a rotary evaporator to ca. 10 ml, and was them diluted with 100 ml of absolute ethanol to produce a pale yellow precipitate. The precipitate was isolated by filtration, and the solid isolated was reprecipitated again from 5 ml of acetonitrile solution by adding 150 ml of ethanol. The pale yellow powder was filtered off, washed with absolute ethanol and air-dried. Yied: 0.98g (57 %). Anal. Calcd for $C_{44}H_{83}N_2O_{39}P_3S_2W_{11}$: C, 15.3; H, 2.41; N, 0.81; P, 2.70; W, 58.8. Found: C, 14.8; H, 2.37; N, 0.75; P, 2.91; W, 59.4%.

#### 2.2.2 $[Bu_4N]_3H[PhP(S)]_2SiW_{11}O_{39}$ **2**

This compound was similarly synthesized from $[Bu_4N]_4KH_3SiW_{11}O_{39}$ (0.8 mmol) and $PhP(S)Cl_2$ (1.6 mmol) as above described. Yield: 1.95 g (65 %). Anal Calcd for $C_{60}H_{119}N_3O_{39}P_2S_2SiW_{11}$: C, 19.60; H, 3.23; N, 1.14; P, 1.68; Si, 0.76; W, 55.00. Found: C, 19.20; H, 3.12; N, 1.09; P, 1,74; Si, 0.72; W, 55.70%.

#### 2.2.3 $[Bu_4N]_3H[PhP(S)]_2GeW_{11}O_{39}$ **3**

$K_6Na_2GeW_{11}O_{39}\cdot 13H_2O$ (2.54 g, 0.8 mmol) and $Bu_4NBr$ (1.29 g, 4 mmol) were suspended in MeCN (25 ml), and an acetonitrile solution of $PhP(S)Cl_2$ (1.6 mmol in 10 ml of MeCN) was added dropwise under vigorous stirring, and the mixture was stirred for 48 h at room temperature. After separation of a solid, the yellow compound $[Bu_4N]_3H[PhP(S)]_2GeW_{11}O_{39}$ was obtained by evaporation of the resulting solution in a rotary evaporator. The crude compound was recrystallized from acetionitrle. Yield 1.1 g (37 %). Anal. Calcd for $C_{60}H_{119}GeN_3O_{39}P_2S_2W_{11}$: C, 19.30; H, 3.19; Ge, 1.96; N, 1.13; P, 1.66; W, 54.30. Found: C, 19.10; H, 3.04; Ge, 1.87; N, 1.08; P, 1.74; W, 55.70%.

2.2.4 $[Bu_4N]_4H[PhP(S)]_2BW_{11}O_{39}$ **4**

$K_7NaHBW_{11}O_{39}\cdot 13H_2O$ (1.59 g, 0.5 mmol) and Bu $_4NBr$ (0.81 g, 2.5 mmol) were suspended in MeCN (25 ml), and an acetonitrile solution of $PhP(S)Cl_2$ (1.0 mmol in 10 ml of MeCN) was added dropwise under vigorous stirring, and the mixture was stirred for 48 h at room temperature. After separation of a white solid, the resulting red solution was concentrated to ca. 10 ml in a rotary evaporator, and was then diluted with 150 ml of absolute ethanol to produce a red brown precipitate. The red brown precipitate was isolated by filtration, and the solid isolated was reprecipited again from 5 ml of acetonitrile solution by adding 100 ml of absolute ethanol to give 1.31 g (67 %) red brown powder. Anal. Calcd for $C_{76}H_{155}BN_4O_{39}P_2S_2W_{11}$: C, 22.80. H, 3.84; B, 1.75; N, 1.40; P, 1.55; W, 50.60. Found: C, 22.40; H, 3.61; B, 1.62; N, 1.35; P, 1.63; W, 51.40%.

2.2.5 $[Bu_4N]_4K[PhP(S)]_2GaW_{11}O_{39}$ **5**

This compound was similarly prepared from $K_9GaW_{11}O_{39}\cdot 13H_2O$ (2.64 g, 0.8 mmol) and $PhP(S)Cl_2$ (1.6 mmol) as above described. Yield: 1.76 g (55%). Anal. Calcd for $C_{76}H_{154}GaKN_4O_{39}P_2S_2W_{11}$: C, 23.30; H, 3.97; Ga, 0.28; N, 1.43; P, 1.59; W, 51.80. Found: C, 23.10; H, 3.41; Ga, 0.25; N, 1.34; P, 1.64; W, 53.20%.

The preparation of $C_6H_{11}P(S)$ derivatives were carried out by following a procedure strictly similar to that used for PhP(S) derivatives using $C_6H_{11}P(S)Cl_2$.

2.2.6 $[Bu_4N]_2H[C_6H_{11}P(S)]_2PW_{11}O_{39}$ **6**

Yield: 60 %. Anal. Calcd for $C_{44}H_{89}N_2O_{39}P_3S_2W_{11}$: C, 15.30; H, 2.75; N, 0.81; P, 2.69; W, 58.60. Found: C, 14.50; H, 2.34; N, 0.72; P, 2.75; W, 53.20%.

2.2.7 $[Bu_4N]_3H[C_6H_{11}P(S)]_2SiW_{11}O_{39}$ **7**

Yield: 60 %. Anal. Calcd for $C_{60}H_{125}N_3O_{39}P_2S_2SiW_{11}$: C, 19.50; H, 3.54; N, 1.14; P, 1.68; Si, 0.76; W, 54.80. Found: C, 18.70; H, 3.42; N, 1.07; P, 1.75; Si, 0.68; W, 56.10%.

2.2.8 $[Bu_4N]_3H[C_6H_{11}P(S)]_2GeW_{11}O_{39}$ **8**

Yield: 51%. Anal. Calcd for $C_{60}H_{125}GeN_3O_{39}P_2S_2W_{11}$: C, 19.30; H, 3.50; Ge, 1.95; N, 1.12; P, 1.66; W, 54.10. Found: C, 19.10; H, 3.23; Ge, 1.86; N, 1.08; P, 1.73; W, 55.60%.

2.2.9 $[Bu_4N]_4K[C_6H_{11}P(S)]_2GaW_{11}O_{39}$ **9**

Yield: 50%. Anal. Calcd for $C_{76}H_{160}GaKN_4O_{39}P_2S_2W_{11}$: C, 22.90; H, 4.20; Ga, 1.76; N, 1.41; P, 1.56; W, 50.90. Found: C, 22.50; H, 3.98; Ga, 1.69; N, 1.37; P, 1.62; W, 51.70%.

2.2.10 $_{}_2$-$[Bu_4N]_5K[PhP(S)]_2P_2W_{17}O_{61}$ **10**

$_{}_2$-$K_{10}P_2W_{17}O_{61}\cdot 17H_2O$ (2.4 g, 0.5 mmol ) and $Bu_4NBr$ (0.97 g, 3 mmol ) were suspended in MeCN (25 ml ), and an acetonitrile solution of $PhP(S)Cl_2$ (1 mmol in 15 ml of MeCN) was added dropwise under vigorous stirring, and the mixture was stirred for 48 h at room temperature. After separation of a white solid, the resulting solution was concentrated to Ca. 10 ml in a rotary evaporator, and was then diluted with 150 ml of anhydrous $Et_2O$ to produce an oily deposit. The turbid supernatant was decanted from the oily deposit, and the deposit was reprecipitated again from 5 ml of acetonitrile solution by adding 150 ml of $Et_2O$ to give 1.80 g (63 %) pale green powder. Anal. Calcd for $C_{86}H_{185}KN_5O_{61}P_4S_2W_{17}$: K, 0.69; P, 2.18; W, 54.80. Found: k, 0.61; p, 2.47; w, 55.60%.

2.2.11 $_{}_2$-$[Bu_4N]_5K[C_6H_{11}P(S)]_2P_2W_{17}O_{61}$ **11**

This compound was similarly prepared from $a_2$-$K_{10}P_2W_{17}O_{61}\cdot 17H_2O$ (2.91 g, 0.6 mmol) and $C_6H_{11}P(S)Cl_2$ (1.2 mmol) as above described. Yield: 2.1 g (62 %). Anal. Calcd for $C_{86}H_{191}KN_5O_{61}P_4S_2W_{17}$: K, 0.69; P, 2.18; W, 54.70. Found: K, 0.73; P, 1.87; W, 53.20%.

2.2.12 A-_-$[Bu_4N]_4H[PhP(S)]_2PW_9O_{34}$ **12**

A-_-$Na_8H[PW_9O_{34}]\cdot 24H_2O$ (2.30 g, 0.8 mmol) and $Bu_4NBr$ (1.5 g, 4.8 mmol) were suspended in MeCN (20 ml), and a MeCN (15 ml) solution of $PhP(S)Cl_2$ (0.24 ml, 1.6 mmol) was added dropwise under vigorous stirring. The solution turned pale yellow, and mixture was stirred overnight under reflux. The solid was filtered off, the resulting blue solution was concentrated to 10 ml in a rotary evaporator, and anhydrous $Et_2O$ (150 ml) was added to produce a blue oily deposit. The turbid supernatant was decanted from the oily deposit, which was redissolved in MeCN (5 ml). This solution was then rediluted with $Et_2O$ (150 ml), and the pale blue sticky solid formed was crushed with a spatula until it became a powder. The solid was filtered off, washed with $Et_2O$, and air-dried to give 1.3 g of pale blue solid. Yield: Ca. 47%. Anal. Calcd for $C_{76}H_{155}N_4O_{34}P_3S_2W_9$: C, 26.20; H, 4.45; N, 1.60; P, 2.70; W, 47.60. Found: C, 26.40; H, 4.40; N, 1.65; P, 2.60; W, 47.80%.

2.2.13 A-_-$[Bu_4N]_4H[C_6H_{11}P(S)]_2PW_9O_{34}$ **13**

This compound was similarly prepared from A-_-$Na_8H[PW_9O_{34}]\cdot 24H_2O$ (2.30 g, 0.8 mmol) and $C_6H_{11}P(S)Cl_2$ (0.27 ml, 1.6 mmol) as above used. Yield 1.26 g (45%). Anal. Calcd for $C_{76}H_{161}N_4O_{39}P_3S_2W_9$: C, 26.10; H, 4.78; N, 1.60; P, 2.66; W, 47.40. Found: C, 26.70; H, 4.98; N, 1.51; P, 2.88; W, 48.10%.

2.3 Physical measurements

IR spectra were recorded on an Alpha Century FT-IR spectrometer in the range 2000-350 $cm^{-1}$ as KBı pellets. $^{31}P$ and $^{183}W$ NMR spectra were recorded at 16.64 MHz on a Unity-400 spectrometer. Chemical shift were referenced to 2M $Na_2WO_4$ in $D_2O$ for $^{183}W$. For $^{31}P$, the chemical shifts were given with respect tc external 85% $H_3PO_4$ in $CD_3CN$.

W, P, Si, Ge, Ga, and B contents were determined using a Leeman corporation inductively coupled plasma (ICP) emission spectrometer while C, H and N contents were determined using a PE-2400 analyser and K was determined by atomic absorption spectroscopy (PE-3030).

2.4 Biological activity studies

The antitumor activity of compounds was tested by the MTT experiment as previously described [5].

The antigerm activity of some compounds was tested using procedures described in literature [14].

### 3. RESULTS AND DISCUSSION

3.1 $^{31}P$ NMR spectra

The $^{31}P$ NMR spectra data for all compounds and several representative spectra are given in Table 1 and Figure 1, respectively. The attachment of thiophosphoryl groups onto the polyoxotungstates surface are demonstrated by the resonance in the $^{31}P$ NMR spectra, which are all distinct from those of $PhP(S)Cl_2$ or $C_6H_{11}P(S)Cl_2$ in the identical medium.

**Table 1 $^{31}$P NMR data of compounds (ppm)**

| Compound | _ |
|---|---|
| $PhP(S)Cl_2$ | 75,6 |
| $C_6H_{11}P(S)Cl_2$ | 101,8 |
| $Bu_4N]_2H[PhP(S)]_2PW_{11}O_{39}$ **1** | 45,7 – 12,8 |
| $[Bu_4N]_3H[PhP(S)]_2SiW_{11}O_{39}$ **2** | 68,9 |
| $[Bu_4N]_3H[PhP(S)]_2GeW_{11}O_{39}$ **3** | 68,6 |
| $[Bu_4N]_4H[PhP(S)]_2BW_{11}O_{39}$ **4** | 71,7 |
| $[Bu_4N]_4K[PhP(S)]_2GaW_{11}O_{39}$ **5** | 68,2 |
| $[Bu_4N]_2H[C_6H_{11}P(S)]_2PW_{11}O_{39}$ **6** | 92,8 – 13,4 |
| $[Bu_4N]_3H[C_6H_{11}P(S)]_2SiW_{11}O_{39}$ **7** | 32,5 |
| $[Bu_4N]_3H[C_6H_{11}P(S)]_2GeW_{11}O_{39}$ **8** | 91,6 |
| $[Bu_4N]_4K[C_6H_{11}P(S)]_2GaW_{11}O_{39}$ **9** | 80,4 |
| $\alpha$-A-$[Bu_4N]_4H[PhP(S)]_2PW_9O_{34}$ **12** | 16,1 – 12,2 |
| $\alpha$-A-$[Bu_4N]_4H[C_6H_{11}P(S)]_2PW_9O_{34}$ **13** | 32,9 – 11,9 |
| $\alpha_2$-$[Bu_4N]_5K[PhP(S)]_2P_2W_{17}O_{61}$ **10** | 15,3 – 10,9 – 12,5 |
| $\alpha_2$-$[Bu_4N]_5K[C_6H_{11}P(S)]_2P_2W_{17}O_{61}$ **11** | 90,3 – 10,5 – 12,8 |

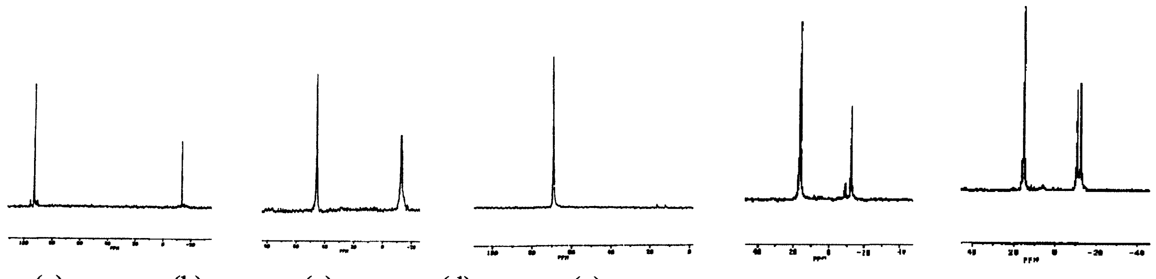

(a) (b) (c) (d) (e)

(a)$[Bu_4N]_2H[C_6H_{11}P(S)]_2PW_{11}O_{39}$ **6**

(b)$[Bu_4N]_2H[PhP(S)]_2PW_{11}O_{39}$ **1**

(c)$[Bu_4N]_3H[PhP(S)]_2SiW_{11}O_{39}$ **2**

(d)A-$\alpha$-$[Bu_4N]_4H[PhP(S)]_2PW_9O_{34}$ **12**

(e) $\alpha_2$-$[Bu_4N]_5K[PhP(S)]_2P_2W_{17}O_{61}$ **10**

**Fig. 1 $^{31}$P NMR spectra of compounds**

The $^{31}P$ NMR spectra of compounds 1, 6, 12 and 13 exhibit two lines with a relative intensity of 2:1, indicating that there are two nonequivalent phosphorus environments. The high-frequency resonances are attributed to the thiophosphoryl group, and the low-frequency single of relative intensity 1 are assigned to the central $PO_4$ unit of the polyoxotungstates portion. The relative intensity indicates a ratio of two RP(S) groups per polyoxometalate, which is consistent with the results of the chemical analysis.

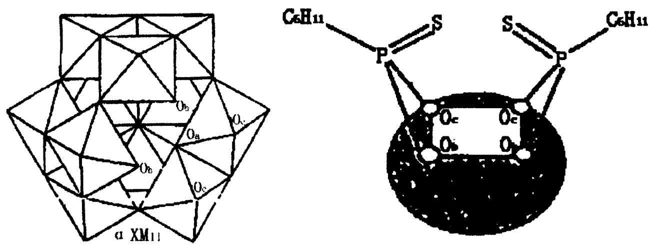

**(a) (b)**

**Fig.2 Schematic representation of $[C_6H_{11}P(S)_2]XW_{11}O_{39}^{n-}$**

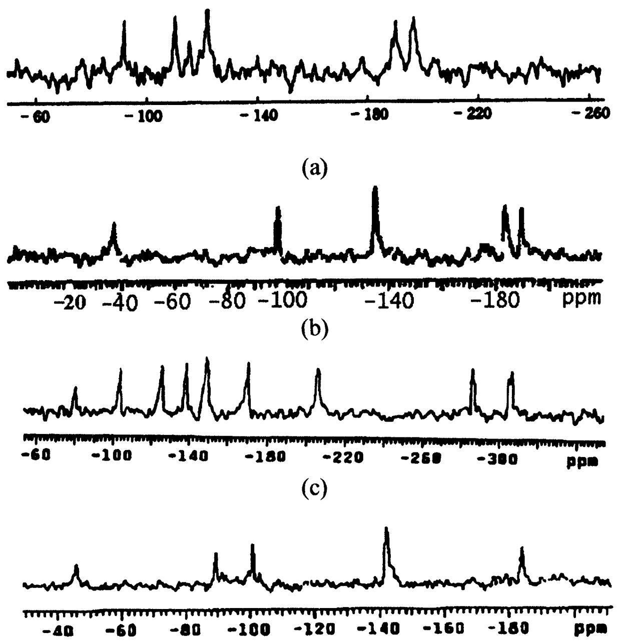

(a) $[Bu_4N]_4H[PhP(S)]_2BW_{11}O_{39}$(4)

(b) A-α-$[Bu_4N]_4H[PhP(S)]_2PW_9O_{34}$ (12)

(c) A-α-$[Bu_4N]_4H[C_6H_{11}P(S)]_2PW_9O_{34}$ (13)

(d) $[Bu_4N]_5K[C_6H_{11}P(S)]_2P_2W_{17}O_{61}$(11)

**Fig. 3 $^{183}$W NMR spectra of compounds**

As for the compounds derived from $P_2W_{17}O_{61}^{10-}$ anion, their $^{31}$P NMR spectra present three lines with a relative intensity ratio of 2:1:1, indicating that there are three non-equivalent phosphorus environments in the complexes. The high-frequency resonance is assigned to the organothiophosphoryl groups. The occurrence of two equal peaks in the low-frequency region shows that the half-anions of $\alpha_2$-$P_2W_{17}$ are not identical. P (1) is the phosphorus atom closest to the site of substitution. P (2) is that remote from the substitution site. It is worth noting that the chemical shift of the phosphorus atom of the unperturbed $PW_9$ half-anion is practically constant; it does not depend upon any change (hole or substitution) that may occur in the other half-anion.

The $^{31}$P NMR spectra of the title compounds show only single line at upfield, indicating that the model of attachment of two organic groups to the lacunary anions are equivalent. A heteropolyanion with a Keggin structure becomes the Cs lacunary polyanion $XM_{11}O_{39}^{n-}$ after losing one heavy atom and its terminal, which contains three $W_3O_{13}$ triads and one $W_2O_{10}$ diad. These anions have a hole surrounded by five oxygen atoms, one Oa, two Ob and two Oc (see Fig.2). When two double-bonded phosphoryl groups each bridges two of the five oxygen atoms that define the hole in the lacunary polyanion, there are two possibilities, i.e. the groups can bridge the oxygens such that they are either unequivalent or equivalent. The single resonance in the $^{31}P$ NMR spectra indicates that the mode of attachment of the organic groups to the lacunary anion is equivalent, i.e. each organic group is connected to two W atoms belonging to a triad and a diad, respectively.

3.2 $^{183}W$ NMR spectra

The $^{183}W$ NMR spectra of compounds 4, 11, 12, 13 and chemical shifts of some compounds are shown in Figure 3 and Table 2, respectively.

**Table 2 $^{183}W$ NMR data of compounds (ppm)**

| Compound | | | | | | |
|---|---|---|---|---|---|---|
| $[Bu_4N]_4H[PhP(S)]_2BW_{11}O_{39}$ | -92,3(2) | -111,4(2) | -116,1(1) | -123,2(2) | -191,5(2) | -197,2(2) |
| $[Bu_4N]_3H[PhP(S)]_2SiW_{11}O_3$ | -21,5(1) | -50,5(2) | -88,4(2) | -121,2(2) | -148,2(2) | -164,4(2) |
| A-α-$[Bu_4N]_4H[PhP(S)]_2PW_9O_{34}$ | -36,9(1) | -98,9(2) | -135,2(2) | -183,7(2) | -189,9(2) | |
| A-α-$[Bu_4N]_4H[C_6H_{11}P(S)]_2PW_9O_{34}$ | -45,9(1) | -89,6(2) | -101,1(2) | -142,4(2) | -184,3(2) | |
| $[Bu_4N]_4K[PhP(S)]_2GaW_{11}O_{39}$ | -62,5(2) | -70,9(1) | -96,3(2) | -99,1(2) | -146,4(2) | -147,9(2) |
| $[Bu_4N]_3H[PhP(S)]_2SiW_{11}O_{39}$ | -79,3(2) | -98,2(1) | -106,7(2) | -159,6(2) | -196,4(2) | -198,1(1) |

The $^{183}W$ NMR spectra of organophosphoryl derivatives of Keggin-type polyoxoanions consist of six peaks, establishing that all species have Cs symmetry in solution.

The $^{183}W$ NMR spectra of compounds 12, 13 consist of five peaks of relative intensity 1:2:2:2:2. In the Well-Known Keggin structure, all the tungsten is identical as shown by a single resonance in the $^{183}W$ NMR spectra. Removal of three $WO_6$ groups reduces the symmetry of the anion from Td to $C_{3v}$ and the expected two-peak pattern is obtained in the $^{183}W$ NMR spectrum. The five-line $^{183}W$ NMR spectra of compounds indicate a lowering of the symmetry of the tungstophosphate framework from $C_{3V}$ to Cs through the attachment of organothiophosphoryl groups. This feature was observed for RPO derivatives of trivacant tungstophosphate [4].

The $^{183}W$ NMR spectrum of compound 11 in $CH_3CN$-$CD_3CN$ consists of nine peaks in the ratio 1:2:2:2:2:2:2:2:2. This pattern confirms a molecule of Cs symmetry as would be found by substitution in the "cap" position of the $[_{2}\text{-}P_2W_{17}O_{61}]^{10-}$ isomer.

**Table 3 Diameter of mycelia block and inhibitory effect against F. graminearum for compounds**

| Compound | 100ppm | | 50ppm | | 20ppm | |
|---|---|---|---|---|---|---|
| | Diameter (mm) | Inhibitory rate (%) | Diameter (mm) | Inhibitory rate (%) | Diameter (mm) | Inhibitory rate (%) |
| Control group | 26.50 | - | 26.50 | - | 26.50 | - |
| $[Bu_4N]_4H[PhP(S)]_2BW_{11}O_{39}$ | 24.00 | 9.43 | 30.00 | - | 31.00 | - |
| $[Bu_4N]_3H[PhP(S)]_2SiW_{11}O_{39}$ | 20.75 | 21.69 | 24.50 | 7.55 | 29.25 | - |
| $[Bu_4N]_4K[PhP(S)]_2GaW_{11}O_{39}$ | 26.67 | - | 31.50 | - | 32.00 | - |
| $[Bu_4N]_4H[PhP(S)]_2PW_9O_{34}$ | 32.76 | - | 33.50 | - | 35.67 | - |
| $[Bu_4N]_5K[PhP(S)]_2P_2W_{17}O_{61}$ | 17.17 | 35.21 | 19.75 | 25.47 | 20.83 | 21.39 |
| $[Bu_4N]_3H[PhCH_2P(O)]_2SiW_{11}O_{39}$ | 31.75 | - | 32.67 | - | 35.50 | - |
| $[Bu_4N]_4H[C_6H_{11}P(O)]_2PW_9O_{34}$ | 32.76 | - | 33.50 | - | 35.67 | - |
| $[Bu_4N]_5K[PhP(S)]_2P_2W_{17}O_{61}$ | 17.17 | 35.21 | 19.75 | 25.47 | 20.83 | 21.39 |
| $[Bu_4N]_3H[PhCH_2CH_2P(O)]_2SiW_{11}O_{39}$ | 31.75 | - | 32.67 | - | 35.50 | - |
| $[Bu_4N]_4H[C_6H_{11}P(O)]_2PW_9O_{34}$ | 23.25 | 12.26 | 24.75 | 6.60 | 27.22 | - |
| $[Bu_4N]_4H[PhP(O)]_2BW_{11}O_{39}$ | 17.67 | 33.32 | 19.50 | 26.42 | 23.00 | 13.21 |
| $[Bu_4N]_3H[C_6H_{11}P(S)]_2SiW_{11}O_{39}$ | 23.50 | 11.32 | 25.50 | 3.77 | 25.50 | 3.77 |
| $[Bu_4N]_5K[PhP(O)]_2P_2W_{17}O_{61}$ | 21.50 | 18.87 | 21.50 | 18.87 | 26.33 | - |
| $[Bu_4N]_5K[PhP(O)]_2P_2W_{17}O_{61}$ | 15.50 | 41.51 | 19.37 | 34.45 | 19.50 | 26.41 |

3.3 Biological activity of some of organophosphoryl polyoxotungstates

Fusarium graminearum causing rice seedling blight and root rot was used in the antigerm activity experiments. The antigerm activity was tested by mycelia block method. Briefly, the test compounds were dissolved in DMSO and diluted to give 10, 20, 50 or 100 times solution, then were diluted with PDA medium to give a final concentration of 100, 50 or 20 ppm, respectively. The germ was incubated in PDA medium for one week, then the mycelia block with 6 mm of hole diameter were added to PDA culture medium contain various amounts of test compounds, and incubated for 8 days at 28° in an incubator. Every test was repeated three times. The antigerm effect of the compounds was judged by the size of diameter of mycelia block grown in medium of various compounds compared to the control. The diameter of mycelia block and inhibitory rate for some of organothiophosphoryl polyoxotungstates and together with compounds reported previously are listed in Table 3.

The antitumor activity of some compounds was tested by the MTT method. The experimental results showed that the title compounds did not exhibit higher antitumor activity as indicated in Table 4.

**Table 4 Inhibitory effects of some compounds on two tumor cell lines in vitro**

| | HL-60 | | B16 |
|---|---|---|---|
| compound | Dose/_g·ml$^{-1}$ | Inhibitory effect (% | Inhibitory effect (%) |
| | 6.39 | 31.82 | 31.35 |
| | 12.79 | 39.21 | 41.65 |
| $[Bu_4N]_4H[PhP(S)]_2GaW_{11}O_{39}$ | 25.58 | 46.86 | 47.83 |
| | 51.15 | 53.12 | 49.25 |
| | 102.3 | 60.90. | 58.17 |
| | 5.43 | 21.45 | 13.90 |
| | 10.85 | 30.06 | 17.83 |
| $[Bu_4N]_5K[PhP(S)]_2P_2W_{17}O_{61}$ | 21.70 | 34.86 | 19.64 |
| | 43.40 | 36.77 | 30.42 |
| | 86.80 | 39.89 | 32.51 |
| | 6.31 | 20.07 | 29.18 |
| | 12.63 | 25.00 | 31.58 |
| $[Bu_4N]_3H[PhP(S)]_2SiW_{11}O_{39}$ | 25.25 | 29.59 | 36.36 |
| | 50.50 | 35.12 | 39.26 |
| | 101.00 | 38.51 | 43.23 |

### ACKNOWLEDGEMENTS

This work was supported by the National Science Foundation of China (no 20171001).

### REFERENCES

[1] J. T. Rhule, C. L. Hill, D. A. Judd, *Chem. Rev.*, **98** (1998) 327.
[2] W. H. Knoth, P. J. Domaille and R. D. Farlee, *Organometallics,* **4** (1985) 62.
[3] G. S. Kim, K. S. Hagen and C. L. Hill, *Inorg. Chem.*, **31** (1992) 5316.
[4] C. R. Mayer, R. Thouvenot, *J. Chem. Soc., Dalton Trans.*, (1998) 7; *Inorg. Chem.*, **38** (1999) 6152.
[5] X. H. Wang, J. F. Liu, Y. G. Chen, Q. Liu, J. T. Liu and M. T. Pope, *J. Chem. Soc., Dalton Trans.*, (2000) 1139.
[6] X. H. Wang, H. C. Dai and J. F. Liu, *Polyhedron,* **18** (1999) 2293.
[7] X. H. Wang, H. C. Dai and J. F. Liu, *Transition Met. Chem.*, **24** (1999) 600.
[8] Z. G. Sun, Q. Liu and J. F. Liu, *Polyhedron,* **19** (2000) 125.
[9] W. T. Tao and X. Y. Li, Modern Chemical Reagent, Chemical Industry press, (1992) 315 (Chinese).
[10] R. Q. Huang, H. L. Wang and J. Zhou, Preparation of Organic Midbody Compound (Chinese).
[11] C. Brevard, R. Schimpt, G. Tourne and C. M. Tourne, *J. Am. Chem. Soc.*, **105** (1983) 7059.
[12] N. Haraguchi, Y. Okaue, T. Isobe and Y. Matsuda, *Inorg. Chem.*, **33** (1994) 1015.
[13] R. Massart, R. Contant, J. M. Fruchart, J. P. Ciabrini and M. Fournier, *Inorg. Chem.*, **16** (1977) 2916.
[14] Z. D. Fang, Investigation methods of plant, Agriculture Publishers, Beijing (1997) (Chinese).

**Received: January 29, 2002 – Accepted: February 19, 2002 – Accepted in publishable format: February 20, 2002**

